# Time of Birth and the Risk of Adverse Maternal and Neonatal Outcomes—A Retrospective Cohort Study

**DOI:** 10.3390/jcm13102952

**Published:** 2024-05-17

**Authors:** Anat Schwartz, Shiri Shinar, Amit Iton-Schwartz, Ronella Marom, Dror Mandel, Ayelet Dangot, Ariel Many

**Affiliations:** 1 Department of Obstetrics and Gynecology, Lis Maternity Hospital, Tel Aviv Sourasky Medical Center, Tel Aviv 6997801, Israel; anat.schwarttz@sheba.health.gov.il (A.S.); ayeletd@tlvmc.gov.il (A.D.); many@tauex.tau.ac.il (A.M.); 2 Faculty of Medical and Health Sciences, Tel Aviv University, Tel Aviv 6997801, Israel; amitito@clalit.org.il (A.I.-S.); ronellam@tlvmc.gov.il (R.M.); drorm@tlvmc.gov.il (D.M.); 3 Department of Obstetrics and Gynecology, Mount Sinai Hospital, Toronto, ON M5G 1X5, Canada; 4 Ontario Fetal Center, Maternal-Fetal Medicine, Mount Sinai Hospital, Department of Obstetrics and Gynaecology, University of Toronto, Toronto, ON M5S 1A1, Canada; 5 Department of Neonatology, Lis Maternity and Women’s Hospital, Tel Aviv Sourasky Medical Center, Tel Aviv 6997801, Israel

**Keywords:** term pregnancy, time of birth

## Abstract

**Objectives:** To determine whether in a labor floor housed continuously by senior physicians the risk of adverse maternal and neonatal outcome is affected by time of delivery. **Methods:** This retrospective cohort study, conducted at a tertiary medical center, assessed singleton term deliveries from 1 January 2011 to 30 January 2020. Participants were categorized based on delivery timing, correlating with nursing shifts, to evaluate perinatal outcomes. The primary endpoint included adverse maternal outcomes such as emergency Cesarean section, anal sphincter injuries, blood product transfusions, and postpartum surgeries (laparotomy/laparoscopy). Secondary outcomes focused on neonatal health indicators, including low Apgar scores, ICU admissions, respiratory issues, extended hospital stays, and neurological complications. **Results:** 87,863 deliveries were available for analysis with equal distribution during the day. The risk of adverse composite maternal outcome was highest during the evening (aOR 1.25, 95% CI 1.18–1.32) and lowest during the night (aOR 0.94, 95% CI 0.88–0.99) compared to daytime deliveries. This difference was primarily driven by the highest rate of emergency CD in the evening. Neonatal outcomes were comparable, except for length of stay > 5 days, which was more frequent among newborns delivered during the evening and night shifts compared to the morning shift (aOR 1.19, 95% CI 1.07–1.33 and aOR 1.17, 95% CI 1.05–1.31, respectively). **Conclusions:** In term pregnancies, the evening shift is associated with the highest risk of adverse maternal and neonatal outcomes despite physician seniority.

## 1. Introduction

The association between time of delivery and maternal and neonatal adverse outcome in term pregnancies is inconclusive [1,2,3,4,5,6,7,8]. Long working hours, shift handovers, staff seniority and weekdays vs. weekends deliveries are all potential causal factors for adverse perinatal outcomes [3,9,10,11,12].

Previous studies on the effect of day versus night shifts on adverse maternal outcomes have demonstrated conflicting findings. A study of 1,475,593 singleton deliveries found that birth between 11 p.m. and 7 a.m. is an independent risk factor for severe maternal morbidity [3]. Similar findings were also demonstrated in term nulliparous women, who exhibited a higher risk of Cesarean delivery and operative vaginal delivery at night. In contrast [4], a study of 11 million births found that the composite maternal adverse outcome was similar among the three different provider time shifts (7 a.m.–3 p.m. vs. 3–11 p.m. and 11 p.m.–7 a.m.) [6]. This finding was also supported by a study of spontaneous singleton deliveries after 34 weeks, that demonstrated no differences in maternal and perinatal outcomes across the hours of the day, aside from a higher rate of third- and fourth-degree tears in the evening [13]. Interestingly, when assessing maternal experience defined according to a visual analogue scale, giving birth during the evening led to impaired childbirth experiences as reported by the mom compared to other times of the day, possibly due to a negative experience related to labor induction [14].

The effect of time of delivery on term neonatal outcomes is also debatable as previous studies have shown inconsistent findings. While one study of 34,000 births did not demonstrate any significant differences in neonatal morbidity and mortality when stratified by time of day of delivery [2], two other large studies found that the nighttime was the most vulnerable period with the highest risk for adverse neonatal events [8,15]. Yet another large study of 11 million term singleton pregnancies found that the rate of neonatal adverse outcomes was marginally but significantly higher when the delivery occurred during the evening (3–11 p.m.) and the night (11 p.m.–7 a.m.) shifts, when compared with the morning shift (7 a.m.–3 p.m.) [6]. Similarly, in a study of 1,048,957 term singleton term births, the risk of severe unexpected neonatal morbidity was significantly higher when the delivery occurred during evening and night shifts [7].

The conflicting existing literature may be related to different practices across various labor wards and changes in staffing seniority between day and night. Therefore, the aim of our study was to determine whether the risk for maternal and neonatal adverse outcome is affected by the time of delivery in a high-volume center, where senior obstetricians and anesthesiologists are always present on the labor floor regardless of the time of day.

## 2. Materials and Methods

This was a retrospective cohort study of 87,863 term singleton births from 1 January 2011 to 31 June 2020, at a single tertiary university affiliated medical center. We excluded elective Cesarean deliveries (CD), multifetal gestations, stillbirth prior to labor onset, pregnancy terminations, and pregnancies complicated by major fetal anomalies. Our cohort was divided into three groups by the time of delivery according to the standard nursing shifts in our hospital–7:00 a.m. to 2:59 p.m., 3:00 p.m. to 10:59 p.m., and 11:00 p.m. to 6:59 a.m. The physician standard shifts in our institute are divided into a morning shift (7:00 a.m. to 3:30 p.m.), supervised by two senior attending physicians, with at least one having more than five years’ experience as a senior obstetrician, and a night shift (3:00 p.m. to 7:00 a.m.), attended by five residents in different stages of their training, and one supervising junior attending physician, with up to five years of seniority, but not necessarily obstetrics oriented. Low risk labors are routinely managed by midwifes, who also deliver the neonate. Induction of labor (IOL) has two major protocols—cervical ripening with prostaglandins (specifically PGE2 slow release via vaginal tablets or gel and rapid release with cervidil), or oxytocin with/without double lumen cervical foley balloon. As our labor and delivery room is very busy with a high volume of births, and is part of the public medical system, induction initiation may take place at any time throughout the day and night based on availability. All data regarding maternal demographic characteristics, pregnancy and delivery outcomes, and neonatal outcomes were extracted from computerized delivery room logbooks and neonatal intensive care unit databases that are updated in real time at our institution.

The primary outcome was a composite adverse maternal outcome, which comprised of the occurrence of an emergency CD, obstetric anal sphincter injuries (OASI), intra-and-post-partum blood product transfusions, and postpartum explorative laparotomy/laparoscopy performed within the postpartum hospitalization. Secondary outcomes were individual maternal outcomes and neonatal outcomes. Maternal outcomes included second and third stage duration, non-elective CD, assisted vaginal delivery, episiotomy, perineal tears and anal sphincter injury, manual revision of the uterine cavity, treatment with blood products, post-partum length of hospitalization, and explorative abdominal surgery during post-partum hospitalization.

Neonatal outcomes included an Apgar score < 7 at 5 min, neonatal hospital length of stay, neonatal intensive care unit (NICU) admission, respiratory complications [including apnea, transient tachypnea of the newborn (TTN) and respiratory distress syndrome (RDS), pneumothorax and meconium aspiration syndrome], and respiratory support [including environmental oxygen supplementation, continuous positive air pressure (CPAP), and invasive ventilation]. Composite adverse neonatal outcome was defined as birth trauma (comprised of neonatal scalp or bony injuries), NICU admission, intraventricular hemorrhage (IVH), necrotizing enterocolitis (NEC), subgaleal hemorrhage, hypoxic-ischemic encephalopathy (HIE), neonatal convulsions, and cerebral cooling. Additionally, the composite of adverse neurological outcome, comprising of a subgaleal hemorrhage, IVH, HIE, convulsions, and neonatal whole-body cooling, was analyzed.

Newborns or women with more than one adverse outcome were counted once when formulating the composites.

### Statistical Analysis

Categorical variables were summarized as frequencies and percentages. Continuous variables were evaluated for normal distributions using a histogram and reported as the median and inter-quartile range (IQR) or mean and standard deviation (SD). Categorical variables were compared using the chi-square test, and continuous variables were compared using analyses of variances (ANOVA), and the Kruskal–Wallis test or Mann–Whitney test. Multivariable logistic regression was applied to study the association between work shifts and the study’s outcome after controlling for potential confounders. All statistical tests were two sided and *p* < 0.05 was considered as statistically significant. SPSS software was used for all statistical analyses (IBMS state for windows, version 27, IBM Corp, Armonk, NY, USA, 2020).

The study was conducted according to the guidelines of the Declaration of Helsinki and approved by the Institutional Review Board (The Tel-Aviv Sourasky medical center IRB 0284-08-TLV, date of approval 1 January 2018).

## 3. Results

**Figure 1:**
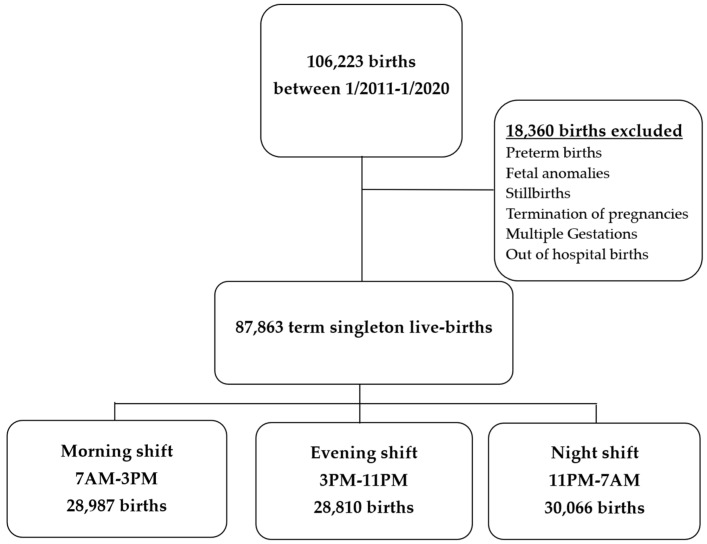
Flowchart of the total number of births in our institution between 2011–2020.

During the nine-year study period, 87,863 pregnancies met inclusion criteria and were available for analysis (Figure 1). A total of 28,987 (33%) women delivered during the morning shift (7 a.m.–3 p.m.), 28,810 (32.8%) delivered during the evening shift (3 p.m.–11 p.m.), and 30,066 (34.2%) delivered during the night shift (11 p.m.–7 a.m.). The mean gestational age at delivery was 39^5/7^ weeks (±1.1 weeks), with no significant difference between the groups (Table 1).

Primiparous women and those with a history of one previous Cesarean delivery (CD) were more likely to deliver during the evening shift (46.3%, and 4.2%, respectively) than during the night shift (40.8%, and 3.3%, respectively) and morning shift (44.3% and 3.9%, Table 1). The incidence of deliveries following spontaneous onset of labor was lowest during the evening shift (75.2%) and highest during the morning shift (80.8%, *p* < 0.01).

The evening shift was characterized by the highest rate of emergency CD, while the night shift—by the lowest rate (13% versus 9.2%, respectively, *p* < 0.01). As for the distribution of certain CD indications, while there was no difference in distribution of CD for non-reassuring fetal heart status (NRFHS) or failed vacuum extraction, the incidence of CD during the second stage of labor was lowest during the night shift in comparison to the morning and evening shifts (9.6% vs. 12.4%, *p* = 0.03). Additionally, the incidence of CD under general anesthesia was lowest in the morning shift, when compared to the evening and night shifts (0.9% vs. 1.2% and 1.3%, respectively, *p* < 0.01, Table 2).

Vacuum assisted vaginal delivery was less frequent during the night shift (6.9%) in comparison to the morning and evening shifts (7.5% and 7.9%, respectively; *p* > 0.01). Women who delivered during the evening shift had the highest incidence of composite adverse maternal outcome while those who delivered during the night shift had the lowest incidence (15.4% versus 11.6%, respectively, *p* < 0.01).

Of all neonatal outcomes assessed, the only outcome which differed between the three time periods was neonatal hospital length of stay of greater that five days, which was more frequent among newborns delivered during the evening and night shifts (3.6% and 3.7%, respectively) compared to the morning shift (3.1%, *p* < 0.01). Moreover, there was no difference in the composite adverse neonatal outcome or the composite adverse neurological outcome between all three time periods (Table 3). Additionally, the proportion of birthweight ≥ 4000 g during morning, evening and night shifts was comparable (0.049, 0.048, 0.045, respectively, *p* = 0.81).

In multivariable analysis controlling for maternal age, pregestational BMI, weight gain during pregnancy, pregestational and gestational diabetes, previous CD and gestational age at delivery, the composite adverse maternal outcome was highest among women who delivered during the evening shift (aOR 1.25, 95% CI 1.18–1.32) and lowest during the night shift (aOR 0.94, 95% CI 0.88–0.99) when compared to the morning shift (Table 4).

As for neonatal outcomes, a similar multivariable analysis found that prolonged neonatal hospitalization > five days was significantly more common among neonates delivered during the evening and night shifts (aOR 1.19, 95% CI 1.07–1.33, and aOR 1.17, 95% CI 1.05–1.31), when compared to the morning shift.

## 4. Discussion

In this large retrospective cohort study of singleton term pregnancies, we found a similar distribution of deliveries across the three work shifts, with a similar frequency of spontaneous onset of labor. When comparing all three work shifts, the evening shift was characterized by a significantly higher rate of composite adverse maternal outcome, whereas the night shift had the lowest rate. There was no difference in the composite adverse neonatal outcome nor in the composite adverse neonatal neurologic outcome between the three work shifts.

We found a significant increase in the composite outcome during the evening shift as compared to the morning shift and a significant decrease during the night shift as compared to the morning shift. This higher rate of adverse maternal outcome during the evening shift is mostly attributed to a higher rate of emergency CD and a greater risk of relaparotomy during the postpartum hospitalization. Although our study is retrospective and as such does not enable determining causality, we believe this, in part, may be due to the higher incidence of primiparous women delivering in the evening. Interestingly, a large study by Wagner et al., which analyzed over 19 million births, also reported higher rates of primiparous and CD during the evening shifts [16]. The incidence of relaparotomy was not discussed in their study. Furthermore, these findings align with those from a previous study examining over 115,000 births, which identified a peak in the incidence of CD during labor at 9 p.m., a decline towards midnight, and a nadir at 10 a.m. [7]. The lower rate of adverse maternal outcome during the night shift contradicts the findings of Lyndon et al. (2015), that demonstrated the highest severe maternal morbidity during the night shift [3]. The discrepancy between their results and ours may stem from the different outcome definitions employed. In fact, the only variable that defined both severe maternal morbidity and our adverse composite maternal outcome was postpartum return to the operating theatre during hospitalization. Our relatively low adverse outcome rate at night may be related to the physician’s preference to refrain from performing indicated but non-emergency procedures during these hours. For example, physicians may be more reluctant to perform a CD due to protracted labor with normal fetal tracing at night, delaying this until the arrival of the next shift. This in turn results in a higher rate of emergency CD in the morning. Indeed, in our cohort CD for second stage arrest was most common during the morning, reflecting this assumption. Our finding of comparable neonatal outcomes, regardless of time of delivery, contradicts several previous studies [2,4,11,16,17] that reported the night shift and the evening to be most vulnerable but supports the findings of other studies that reported comparable neonatal risk [1]. Gould et al., who analyzed over one million births from uncomplicated term pregnancies in California, found an increased risk of adverse neonatal outcomes during evening and night shifts, as well as on Sundays. However, the data for this extensive study were obtained from a statewide database that includes birth certificates, neonatal discharge records, and maternal discharge information. Consequently, the findings reflect a diverse population from various departments, characterized by a non-uniform availability of in-house neonatologists [2]. 

A possible explanation for the similar rates of adverse events observed throughout the day in our study is continuous house staffing by senior obstetricians and anesthesiologists 24 h a day. This finding is consistent with a previous study by Caughey et al., which was conducted in a similar setting and reported a smaller sample population [1]. Another possible explanation for the lack of difference in neonatal outcomes is that our center has an average of 13,000 deliveries a year. As such, our staff encounters obstetrical complications on a daily basis and is well trained to cope with high-acuity medical situations. Lastly, while pediatric residents are present at the hospital during the evening and night shifts, senior neonatologists are on call from home but are routinely called in cases of foreseen potential adverse neonatal outcomes. Our finding that prolonged neonatal hospitalization was independently associated with evening and nighttime deliveries may be related to these staffing differences.

The strengths of this study include its single center design, its detailed clinical and demographic information and its relatively large sample size of roughly 30,000 deliveries in each of the three arms. This number has sufficient power to detect differences in less common maternal outcomes such as postpartum return to the operating theatre and rarer neonatal outcomes. Prior registry-based studies mostly assessed differences in major and rare maternal and neonatal adverse events such as death, ICU admission and hysterectomy [2,9,12]. We, on the other hand, focused on a broader range of more common morbidities and interventions among women undergoing vaginal and emergency CD, such as assisted vaginal deliveries, OASI, CD in the second stage of labor and blood transfusions. Lastly, our delivery data is recorded in real time, minimizing recall bias.

This study has several limitations well worth noting. The data are observational and cannot demonstrate causation. As in other large cohort studies, some of the associations found in our study have modest RR values and should be considered cautiously according to their clinical relevance [16]. The dichotomous categorization of day versus evening versus night may not fully capture time-dependent differences in hospital-based systems, as each hospital may have individual factors that affect schedules or resources, and some operate with 24-h shifts whereas others with day and night floats. This along with the academic based setting of our tertiary setting may limit generalizability of our results. Lastly, it should be noted that in our institute staffing of the labor floor changes during the weekend. While nursing shifts remain unchanged, physician shifts extend to 26 consecutive hours, covered by a junior attending and five residents, with senior attendings available as backup within 20 min of the hospital. As a separate subanalysis comparing weekends to weekdays revealed similar findings, with no significant change in outcome, we chose not to present it in the current study.

In conclusion, our study highlights that in term singleton pregnancies, adverse maternal outcomes are affected by time of delivery with the most deleterious period being the evening shift, while neonatal outcomes remain unaffected by time of delivery.

## Figures and Tables

**Table 1:** Maternal demographic and obstetric characteristics stratified by time of birth.

Time of Birth	7:00–14:59 (Morning Shift)	15:00–22:59 (Evening Shift)	23:00–6:59 (Night Shift)	*p* Value
Number of births	28,987 (33)	28,810 (32.8)	30,066 (34.2)	
Gestational age at delivery (weeks)	39.7 ± 1.12	39.7 ± 1.14	39.8 ± 1.13	
Maternal age at delivery (years)	32.36 ± 4.88	32.48 ± 4.9	32.51 ± 4.85	<0.01 ^a^
Primiparous	12,826 (44.3)	13,326 (46.3)	12,263 (40.8)	<0.01 ^a,b,c^
Previous one CD	1136 (3.9)	1212 (4.2)	987 (3.3)	<0.01 ^c^
Spontaneous pregnancy	25,241 (90.6)	25,047 (90.3)	26,145 (90.6)	0.40
Pre-gestational BMI (kg/m^2^)	22.79 ± 4.12	22.86 ± 4.11	22.83 ± 4.12	0.04 ^b^
Weight gain in pregnancy (kg)	13.35 ± 5.3	13.38 ± 5.32	13.36 ± 5.26	0.83
Pre-gestational diabetes	127 (0.4)	136 (0.5)	140 (0.5)	0.81
Chronic hypertension	103 (0.4)	117 (0.4)	106 (0.4)	0.48
Gestational Diabetes Mellitus	1229 (4.2)	1400 (4.9)	1343 (4.5)	<0.01 ^b^
Spontaneous onset of labor	23,156 (80.8)	21,106 (75.2)	23,444 (79.1)	<0.01 ^a,b,c^
Composite adverse maternal outcome	3648 (12.6)	4450 (15.4)	3498 (11.6)	<0.01 ^a,b,c^

CD Cesarean Delivery; BMI Basic Metabolic Index. ^a^ Morning shift significantly differs from other shifts. ^b^ Evening shift significantly differs from other shifts. ^c^ Night shift significantly differs from other shifts. Data are presented as n (%) or mean (SD) where appropriate. Categorical data were compared using the chi-square test. Continuous variables were compared using analyses of variances (ANOVA), Kruskal–Wallis test or Mann–Whitney test.

**Table 2:** Maternal outcome stratified by time of birth.

Time of Birth	7:00–14:59 (Morning Shift)	15:00–22:59 (Evening Shift)	23:00–6:59 (Night Shift)	*p* Value
Unscheduled Cesarean deliveries	2801 (9.7)	3757 (13)	2751 (9.2)	<0.01 ^a,b,c^
CD during second stage of labor	138 (12.4)	191 (12.4)	135 (9.6)	0.02 ^c^
CD due to NRFHR	933 (83.9)	1301 (84.3)	1219 (86.6)	0.09
CD under General anesthesia	254 (0.9)	358 (1.2)	386 (1.3)	<0.01 ^a^
CD due to failed vacuum extraction	41 (3.7)	52 (3.4)	53 (3.8)	0.82
Instrumental delivery	2187 (7.5)	2266 (7.9)	2075 (6.9)	<0.01 ^c^
Second stage duration (minutes)	34 (12, 104)	28 (10, 96)	24 (10, 82)	<0.01 ^a,b,c^
Third stage duration (minutes)	13 (10, 17)	12 (10, 16)	13 (10, 17)	<0.01 ^b^
Episiotomy	4689 (16.2)	5112 (17.7)	4399 (14.6)	<0.01 ^a,b,c^
OASI	129 (0.4)	150 (0.5)	123 (0.4)	0.124
Manual revision of uterine cavity	1286 (5)	1332 (5.4)	1461 (5.4)	0.06
Intra-and-post-partum treatment with blood products	480 (1.7)	470 (1.6)	432 (1.4)	0.06
post-partum abdominal surgery during admission	18 ^a,b^ (0.1)	27 ^b^ (0.1)	11 ^a^ (0)	0.02 ^c^
Post-partum length of Hospitalization	2.9 ± 1.15	2.41 ± 1.17	2.74 ± 1.24	<0.01 ^a,b,c^
Composite adverse maternal outcome	3648 (12.6)	4450 (15.4)	3498 (11.6)	<0.01 ^a,b,c^

CD Cesarean Delivery; NRFHR Non reassuring fetal heart rate; OASI Obstetric anal sphincter injury. Composite adverse maternal outcome comprised of treatment with blood products, Obstetric anal sphincter injury (OASI), abdominal surgery during post-partum hospitalization, and emergent CD. ^a^ Morning shift significantly differs from other shifts. ^b^ Evening shift significantly differs from other shifts. ^c^ Night shift significantly differs from other shifts. Data are presented as n (%) or mean (SD) where appropriate. Categorical data were compared using the chi-square test. Continuous variables were compared using analyses of variances. (ANOVA), Kruskal–Wallis test or Mann–Whitney test.

**Table 3:** Neonatal outcome stratified by time of birth.

Time of Birth	7:00–14:59 (Morning Shift)	15:00–22:59 (Evening Shift)	23:00–6:59 (Night Shift)	*p* Value
Apgar score at 5 min < 7	45 (0.2)	52 (0.2)	50 (0.2)	0.28
NICU admission	864 (3)	890 (3.1)	896 (3)	0.67
Neonatal hospitalization > 5 days	850 (3.1)	977 (3.6)	958 (3.7)	<0.01 ^a^
Composite adverse neonatal outcome	937 (3.2)	962 (3.3)	971 (3.2)	0.69
Respiratory complications and respiratory support	279 (1)	286 (1.0)	308 (1.0)	0.07
Composite neurological adverse neonatal outcome	188 (0.6)	188 (0.7)	207 (0.7)	0.80

NICU Neonatal intensive care unit. Composite adverse neonatal outcome comprised of neonatal diagnosis of Intraventricular hemorrhage, Neonatal enterocolitis, Sub-galeal hematoma, respiratory complications (as described below), Hypoxic-ischemic encephalopathy, NICU admission, Convulsions, Birth trauma, Cooling, and/ or intra/post-partum death. Respiratory complications and respiratory support comprised of neonatal diagnosis of Transient tachypnea of the newborn, Respiratory distress syndrome, Meconium aspiration syndrome, Apnea, Pneumothorax, and/ or treatment with environmental oxygen support, Continuous positive airway pressure, and/ or intubation. Composite neurological adverse neonatal outcome comprised of neonatal diagnosis of Sub-galeal hematoma, Intraventricular hemorrhage, Hypoxic-ischemic encephalopathy, Convulsions, and/or Cooling. ^a^ Morning shift significantly differs from other shifts. Data are presented as n (%) or mean (SD) where appropriate. Categorical data were compared using the chi-square test. Continuous variables were compared using analyses of variances. (ANOVA), Kruskal–Wallis test or Mann–Whitney test.

**Table 4:** Multivariate logistic regression of composite adverse maternal outcome and prolonged neonatal hospitalization by time of delivery.

Time of Birth	7:00–14:59 (Morning Shift)	15:00–22:59 (Evening Shift)	23:00–6:59 (Night Shift)
Composite adverse maternal outcome	3648 (12.6)	4450 (15.4)	3498 (11.6)
AOR (95% CI)	ref	1.25 (1.18–1.32)	0.94 (0.88–0.99)
Neonatal hospitalization > 5 days	850 (3.1)	977 (3.6)	958 (3.7)
AOR (95% CI)	ref	1.19 (1.07–1.33)	1.17 (1.05–1.31)

AOR Adjusted odds ratio; CI Confidence interval. Composite adverse maternal outcome comprised of treatment with blood products, Obstetric anal sphincter injury (OASI), abdominal surgery during post-partum hospitalization, and emergent CD. Multivariate models control for maternal age, gestational age at delivery, maternal BMI, weight gain during pregnancy, pregestational and gestational hypertension, pregestational and gestational diabetes, previous Cesarean delivery.

## Data Availability

The data that support the findings of this study are available upon reasonable request from the corresponding author, S.S. The data are not publicly available due to their containing information that could compromise the privacy of the study participants.

## References

[1] Caughey A.B., Urato A.C., Lee K.A., Thiet M.P., Washington A.E., Laros R.K. (2008). Time of delivery and neonatal morbidity and mortality. Am. J. Obstet. Gynecol..

[2] Gould J.B., Qin C., Chavez G. (2005). Time of Birth and the Risk of Neonatal Death. Obstet. Gynecol..

[3] Lyndon A., Lee H.C., Gay C., Gilbert W.M., Gould J.B., Lee K.A. (2015). Effect of time of birth on maternal morbidity during childbirth hospitalization in California. Am. J. Obstet. Gynecol..

[4] Liu L.Y., Miller E.S., Yee L.M. (2019). Association between time of day and performance, indications, and outcomes of obstetric interventions among nulliparous women delivering at term. J. Perinatol..

[5] Stewart J.H., Andrews J., Cartlidge P.H.T. (1998). Numbers of deaths related to intrapartum asphyxia and timing of birth in all Wales perinatal survey, 1993–1995. BMJ.

[6] Gould J.B., Abreo A.M., Chang S.C., Main E.K. (2020). Time of birth and the risk of severe unexpected complications in term singleton neonates. Obstet. Gynecol..

[7] Son M., Lai Y., Bailit J., Reddy U.M., Wapner R.J., Varner M.W., Thorp J.M., Caritis S.N., Prasad M., Tita A.T.N. (2020). Association Between Time of Day and the Decision for an Intrapartum Cesarean Delivery. Obstet. Gynecol..

[8] Yee L.M., McGee P., Bailit J.L., Reddy U.M., Wapner R.J., Varner M.W., Thorp J.M., Leveno K.J., Caritis S.N., Prasad M. (2019). Daytime Compared With Nighttime Differences in Management and Outcomes of Postpartum Hemorrhage. Obstet. Gynecol..

[9] Stephansson O., Dickman P.W., Johansson A.L.V., Kieler H., Cnattingius S. (2003). Time of birth and risk of intrapartum and early neonatal death. Epidemiology.

[10] Bailit J.L., Landon M.B., Thom E., Rouse D.J., Spong C.Y., Varner M.W., Moawad A.H., Caritis S.N., Harper M., Wapner R.J. (2006). The MFMU Cesarean Registry: Impact of time of day on cesarean complications. Am. J. Obstet. Gynecol..

[11] De Graaf J.P., Ravelli A.C.J., Visser G.H.A., Hukkelhoven C., Tong W.H., Bonsel G.J. (2010). Increased adverse perinatal outcome of hospital delivery at night. BJOG.

[12] Bailit J.L., Landon M.B., Lai Y., Rouse D.J., Spong C.Y., Varner M.W., Moawad A.H., Simhan H.N., Harper M., Wapner R.J. (2008). Maternal-Fetal Medicine Units Network Cesarean Registry: Impact of shift change on cesarean complications. Am. J. Obstet. Gynecol..

[13] Tavares S., Cavaco-Gomes J., Moucho M., Severo M., Mateus M., Ramalho C., Visser G.H.A., Montenegro N. (2017). 24/7 Presence of Medical Staff in the Labor Ward; No Day-Night Differences in Perinatal and Maternal Outcomes. Am. J. Perinatol..

[14] Joensuu J., Saarijärvi H., Rouhe H., Gissler M., Ulander V.M., Heinonen S., Mikkola T. (2021). Maternal childbirth experience and time of delivery: A retrospective 7-year cohort study of 105 847 parturients in Finland. BMJ Open.

[15] Landrigan C.P., Rahman S.A., Sullivan J.P., Vittinghoff E., Barger L.K., Sanderson A.L., Wright K.P., O’Brien C.S., Qadri S., St Hilaire M.A. (2020). Effect on Patient Safety of a Resident Physician Schedule without 24-Hour Shifts. N. Engl. J. Med..

[16] Wagner S.M., Chen H.Y., Gupta M., Chauhan S.P. (2020). Association of Time of Delivery with Composite Adverse Outcomes in Low-Risk Pregnancies. Obstet. Gynecol..

[17] Reif P., Pichler G., Griesbacher A., Lehner G., Schöll W., Lang U., Hofmann H., Ulrich D. (2018). Do time of birth, unit volume, and staff seniority affect neonatal outcome in deliveries at ≥34 +0 weeks of gestation?. BJOG.

